# Advances in Electronic-Nose Technologies for the Detection of Volatile Biomarker Metabolites in the Human Breath

**DOI:** 10.3390/metabo5010140

**Published:** 2015-03-02

**Authors:** Alphus D. Wilson

**Affiliations:** Southern Hardwoods Laboratory, Center for Bottomland Hardwoods Research, Southern Research Station, USDA Forest Service, P.O. Box 227, Stoneville, MS 38776, USA; E-Mail: dwilson02@fs.fed.us; Tel.: +1-662-686-3180; Fax: +1-662-686-3195

**Keywords:** artificial olfaction, biomarker indicator compounds, breath gas analysis, breathprints, disease diagnostics, electronic aroma detection, e-nose, metabolomics, respiratory gas metabolites, volatile organic compounds

## Abstract

Recent advancements in the use of electronic-nose (e-nose) devices to analyze human breath profiles for the presence of specific volatile metabolites, known as biomarkers or chemical bio-indicators of specific human diseases, metabolic disorders and the overall health status of individuals, are providing the potential for new noninvasive tools and techniques useful to point-of-care clinical disease diagnoses. This exciting new area of electronic disease detection and diagnosis promises to yield much faster and earlier detection of human diseases and disorders, allowing earlier, more effective treatments, resulting in more rapid patient recovery from various afflictions. E-nose devices are particularly suited for the field of disease diagnostics, because they are sensitive to a wide range of volatile organic compounds (VOCs) and can effectively distinguish between different complex gaseous mixtures via analysis of electronic aroma sensor-array output profiles of volatile metabolites present in the human breath. This review provides a summary of some recent developments of electronic-nose technologies, particularly involving breath analysis, with the potential for providing many new diagnostic applications for the detection of specific human diseases associated with different organs in the body, detectable from e-nose analyses of aberrant disease-associated VOCs present in air expired from the lungs.

## 1. Introduction

Traditional methods for the detection and diagnosis of diseases are often invasive and expensive or require time-consuming biological (culturing), microscopic (cell or tissue biopsy) or complex analytical (chemical) tests. Some conventional methods used in clinical diagnoses include many invasive and potentially hazardous biopsy procedures, endoscopy [1], computed tomography [2], DNA marker and homology tests, magnetic resonance imaging (MRI) [3], mammography [4], microbial culture tests, positron emission tomography (PET) [5], serological and other blood tests, ultrasonography [6] and X-ray imaging of other organs [7]. Many of these methods not only present some risks of serious negative side effects, but often are sufficiently painful to discourage patients from participating in preemptive, prophylactic disease-screening procedures. These human-sentiment factors continue to point to the increasing need for improvements in diagnostic methods toward more non-invasive and painless procedures in disease screenings for early diagnosis and for patient examinations and checkups in routine clinical practice.

The search for noninvasive methods of human disease diagnosis have led to the discovery of more rapid, electronic methods of detecting and analyzing complex gaseous mixtures containing volatile organic compounds (VOCs), including metabolites and abnormal chemicals that are specific indicators (biomarkers) of disease, released from the body directly in air expired from the lungs and body cavities or from the headspace of body fluids (blood, serum, sputum, urine, *etc*.) and soft tissue samples collected from sick patients during clinical examinations [8]. Electronic aroma detection (EAD) technologies encompass a wide array of electronic-nose (e-nose)-type technologies with many different gas-detection mechanisms and operating principles [9]. The many types of e-nose instruments range from surface acoustic wave (SAW), quartz crystal microbalance (QMB), metal oxide semiconducting (MOS) and conducting polymers (CP), to the newer DNA-carbon nanotubes [10], and many others [9].

Electronic-nose instruments have many advantages over traditional analytical tools used for chemical analyses, including being less expensive, ease of use and operation without extensive training required, rapid results and response time, quick sensor recovery time, excellent precision, low operating costs, smaller with greater portability and large flexibility in sensor array specificity for selective, specialized applications [9,11,12]. Some disadvantages of e-nose instruments are the inability to identify individual compounds in complex mixtures, sensor arrays sensitive to water vapor, relatively short sensor life, difficulty in measuring analyte concentrations accurately, somewhat lower sensitivity than analytical chemistry instruments and problems of sensor translation [9].

E-nose instruments offer potentially new, non-invasive, cost-effective (potentially inexpensive) approaches to clinical disease diagnosis by means of real-time VOC detection and analysis of exhaled breath samples. Nevertheless, the field of breath analysis research is just beginning and faces many challenges, mainly because the biochemical mechanisms behind the release of disease-related VOCs in the body are largely unknown, and therefore, the correlations between proposed biomarker metabolites and disease are still tenuous and require considerably more investigations.

This review will explore and summarize many of the emerging developments of e-nose technologies for human disease diagnosis, particularly in the more specific and specialized research field of breath analysis or metabolomics. Breath analysis is an area of disease diagnostics that is receiving increasing attention due to the great potential for the simultaneous noninvasive detection and diagnosis of diseases in all parts of the body, based on the production and release of abnormal metabolites (disease biomarkers) from point sources in tissues of individual organs, which are eventually released from the body through the human breath. Breath-associated exogenous biomarkers also are potential indicators of increased risk of future disease development in the body due to short-term or prolonged exposure to toxic or noxious fumes through inhalation.

## 2. Biomarkers of Disease

Disease-related biomarkers (as defined in medicine) are chemicals, usually VOCs, which indicate the presence or severity of a particular disease state or some abnormal physiological condition of an organism. A biomarker may be a chemical substance introduced into an organism (exogenous) or produced within the body (endogenous), which can be detected and measured in the blood, bodily fluids, tissues or human breath, and serves as an indicator of either normal or disease processes in the body. Biomarkers are useful for measuring the progress of disease, evaluating the most effective therapeutic treatments for a particular disease type and establishing long-term susceptibility to specific types of diseases. They are useful in early disease detection and diagnosis, disease prevention, drug target identification, drug response, determining probable effects of treatments on a patient (via risk indicator or predictive biomarkers), establishing if a disease already exists (diagnostic biomarker) and provide indications as to how a particular disease may develop in an individual case regardless of the type of treatment (prognostic biomarker). Predictive biomarkers facilitate the assessment of the most likely responses to a particular treatment type, while prognostic biomarkers show the progression of disease with or without treatment.

The precise definition of disease biomarkers varies somewhat with different researchers, particularly between those in different research fields. Some scientists include external (exogenous) sources of chemicals as biomarkers when they appear to be partially or wholly responsible for causing or inducing disease development in the body; while others limit the term to include only endogenous chemicals, abnormal VOC metabolites produced as a result of the disease process (pathogenesis) and, thus, bio-indicators of a disease condition somewhere within the human body.

### 2.1. Chemical Classes of Disease Biomarkers

The first important consideration in deriving correlations between volatile metabolites and the biochemical processes associated with pathogenesis (disease processes) in the body from which they are derived is to determine which VOCs in the human breath can be considered the result of “normal” physiological processes and distinguish these from the “abnormal” VOCs that are produced as a result of disease processes. More than 2,000 VOCs have been identified in the human breath that can be considered normal [13], but even some of these volatiles can indicate a disease condition (somewhere in the body) if elevated or decreased concentrations are found in the breath. Some of the most common (normal) exhaled breath metabolites found in healthy individuals include the major atmospheric unmodified exogenous gases, such as nitrogen (72% by volume), oxygen (reduced from 21% inhaled to about 15% exhaled), carbon dioxide (about 5%), water vapor (about 6% at saturation) and argon (about 1%) [14]. In addition to these major normal gases, other common endogenous VOCs include ammonia, acetone, ethanol, methanol, propanol, acetaldehyde and isoprene [15,16,17,18,19,20]. Ammonia was found to be a major breath metabolite, measured at a concentration of 833 ppb, followed by acetone (477 ppb), methanol (461 ppb), ethanol (112 ppb), isoprene (106 ppb), acetaldehyde (22 ppb) and propanol (18 ppb) [17,18,19,20].

A wide range of abnormal VOCs, discovered as possible biomarkers of various human diseases, are members of a large diversity of organic chemical classes. Some of the more common chemical classes to which disease biomarker VOCs belong include aliphatic hydrocarbons, aromatic hydrocarbons, alcohols, aldehydes, carboxylic acids (organic acids), esters, ethers, heterocyclic hydrocarbons, ketones, nitriles, sulfides and terpenoids or isoprenoids (terpenes and derivatives) [12]. Different classes of VOCs have many different physicochemical properties that largely determine their distribution, adverse effects and retention in the human body, as well as mechanisms and rates of release from the body in the breath or by other excretory means.

The physicochemical characteristics or chemical nature of disease biomarker VOCs also determine the best or most appropriate methods for detection and identification, either individually using analytical chemistry techniques or in complex mixtures in the human breath, which are best identified as collective aroma patterns, often referred to as breathprints, breath profiles or breath signatures.

### 2.2. Origins of Disease Biomarkers

The types and origins of biomarkers in the human breath are important for determining the sources and potential negative effects associated with the presence of specific VOCs in the body and for evaluating the diagnostic significance of breath biomarkers found (in terms of disease detection in the body), localization of pathogenic effects in specific organs or body compartments and the severity of disease development (as indicators of measurable stages of pathogenesis) or progress of the disease over time. In this way, repeated measures of biomarkers in the breath over time can indicate the rate of disease development and the duration of disease presence in the body.

#### 2.2.1. Physicochemical Characteristics of VOCs

The partition coefficients of biomarker VOCs between blood and air (λ_b:a_) and between fat and blood (λ_f:b_) are used to estimate the equilibrium concentration of VOCs in the blood relative to exhaled air and in fat tissue relative to blood, respectively [12]. These partition coefficients, measured in dimensionless units, e.g., mol L_b_^-1^/mol L_a_^-1^, largely determine the chemical characteristics of breath VOCs and the likelihood that a particular VOC is capable of existing in specific compartments of the body. As a result, lipophilic compounds of higher molecular weights or higher boiling points (BP) may occur at a low concentration (~1–2 ppb) in the exhaled breath, but be indicative of a relatively high concentration in the fat compartment due to lower volatility and greater solubility in blood that hinders out-gassing of these VOCs from the lung airways.

Breath gases with low solubility in blood, mainly nonpolar (λ_b:a_ <10) compounds, undergo pulmonary gas exchange (PGE) almost exclusively in the alveoli, while VOCs that are well soluble in blood, primarily polar (λ_b:a_ >100) compounds, tend to also exchange in the airways of the lungs [21,22,23,24]. Further studies of PGE have shown that VOCs with intermediate λ_b:a_ values between 10 and 100 are involved in exchanges in the airways and alveoli, suggesting that the airways may play a more significant role in PGE than previously believed. The breath VOC profile is influenced by the time of retention of VOCs in the lungs, the physicochemical properties of individual VOCs and their interactions with the various PGE processes [25,26].

#### 2.2.2. Exogenous VOCs

Exogenous VOCs detected in the human breath have received much interest, because the presence of these chemicals may indicate prior exposure of an individual to toxic chemicals and possible carcinogens. Since exogenous VOCs are potentially direct indicators of exposure to disease-causing chemical agents (toxins, noxious or toxic fumes, *etc*.), their detection implies the possibility of adverse effects having occurred to the human body, not only in the form of diseases, but also physical damage to organs that can lead to organ dysfunctions or failures, metabolic disorders and significant tissue damage to lungs, brain and organ systems, particularly the circulatory and nervous systems. The presence of exogenous VOCs in the breath do not necessarily indicate the presence of disease or damage to the body, but only provide clues that disease or damage to organs is more probable and may be present. Therefore, individuals with exposure to these chemical are potentially at higher risk of a higher incidence of disease and are candidates for e-nose breath-analysis screenings to detect early stages of disease.

A large variety of trace exogenous gases exist in ambient air that is taken up via inhalation, skin absorption and through ingestion. The majority of these trace VOCs found in exhaled breath probably originate from exogenous sources and should be distinguished from endogenous compounds [15,27]. Exogenous VOCs can originate from a variety of point sources, such as from building fires, industrial and transportation-related air pollution, municipal air effluents (pollutants), toxic chemical spills and natural pollutants, such as sulfur dioxide and hydrogen sulfide from geothermal sources (volcanoes, hot springs and geysers), eutrophic swamps and bogs and mines open to the atmosphere. Exogenous VOCs are considered highly reactive and typically cause peroxidative damage to DNA, proteins and polyunsaturated fatty acids (PUFA) [12]. The negative impacts of exogenous VOCs on the body can accumulate over many years and result in chronic diseases and cancer [28]. Once exogenous lipophilic toxic VOCs are inhaled and pass from the lungs into the circulatory system, they may be stored within fat compartments of the body and eventually released to cause damage and disease for weeks or months after the initial exposure [29]. Most proposed cancer biomarkers are lipophilic and are reported to be stored in the fat compartment [12].

The levels of exogenous VOCs detected in the breath depend on many factors, including the ambient concentrations in inhaled air, the duration of exposure to ambient air, the solubility and partition coefficients of VOCs into tissues, the mass and fat content of individuals and endogenous concentration relative to blood concentrations. Schubert *et al*. [30] suggested that exhaled concentrations of specific exogenous VOCs cannot be confidently correlated with blood levels of the same compounds when inhaled (ambient) concentrations of exogenous compounds are greater than 5% of the exhaled concentrations. The concept of the alveolar gradient [31], defined as the abundance in breath minus ambient air, does not account for variable VOCs in inhaled background air, as demonstrated in numerous studies.

#### 2.2.3. Endogenous VOCs

The normal endogenous VOCs found in human breath, largely derived from internal tissue sources as a result of human metabolism, such as inorganic gases (e.g., CO, CO2 and NO) and VOCs (isoprene, ethane, pentane, acetone, *etc*.) are detected and measured directly in the breath, while other, typically non-volatile substances (e.g., isoprostanes, peroxynitrite or cytokines) are measured in the breath condensate [32]. These non-volatile substances occur in exhaled breath as aerosol particles.

Endogenous VOCs tend to be less reactive that exogenous VOCs and have a much lower tendency to be stored in the fat compartments of the body (due to their greater volatility). Endogenous VOCs also have a stronger tendency to be dissolved in the blood (high blood solubility due to greater polarity) for easier transport via the blood to the lungs for release through exhalation [33,34,35,36]. The physicochemical characteristics of higher polarity and shorter retention in the body make the endogenous VOCs more conducive to detection in the breath, more rapidly after formation, by use of electronic sensor arrays.

## 3. Specificity of Disease-Associated Biomarkers

Diseases of the human body can arise from several different major mechanisms having origins derived from genetic defects (such as metabolic disorders), short- or long-term exposure to exogenous abiotic toxins or teratogenic chemicals and through biotic agents (pathogens), generally involving a microbe capable of inducing states of pathogenesis by altering the normal biochemical and physiological processes of the host. Each of these disease-causing mechanisms produce and release different types of aberrant chemicals into the body that are characteristic of the particular disease mechanism and the specific microbes involved. Pathogenic microbes themselves also release unique combinations of volatile metabolite biomarkers in the body, which become part of the detectable VOC disease signatures associated with biotic causal agents. Many diseases affect only certain organs or organ systems in the body. This specificity of disease effects on certain organs largely determines the types of aberrant chemicals produced as a result of the specialized biochemical processes that occur in specific organs, the unique physiological pathways of the host that are altered and the peculiar metabolites that result from pathogenesis or altered genetic expressions of defective genes.

The unique mechanisms of disease and resulting abnormal chemicals produced by different types of human diseases result in the production and release of chemicals into affected tissues, regardless of their location in the body, which are picked up by the circulatory system and eventually exchanged and expelled through the lungs. As a consequence, the complex gaseous mixtures released from the lungs through the breath provide biochemical clues (useful for diagnoses) to all of the aberrant biochemical processes occurring simultaneously in the body, including diseases that may occur in any tissues or organs. The general overall health of an individual may, therefore, be measured and monitored on a continuous basis to determine changes in the healthful state of the body as a whole based on the presence or absence of specific biomarker breath compounds that have been associated, through empirical research, with specific diseases. Breath monitoring of patients following treatments can provide indications of the effectiveness of treatments, recovery from disease conditions and indications of the prognoses going forward.

### 3.1. Biomarkers of Metabolic Diseases

Metabolic diseases are physiological disorders, generally caused by inherited genetic defects, which often result in a deficiency or inadequate production of an enzyme or hormone (e.g., insulin) required for normal metabolic activities. Hundreds of different genetic-based metabolic disorders, related to a single missing enzyme, cause various symptoms depending on that enzyme’s job, but usually cause the buildup of toxic metabolites that the deficient enzyme is responsible for converting to other essential products in anabolic or catabolic processes (biochemical pathways).

Because metabolic diseases often involve the production of an excess or deficiency in a specific enzyme needed for the metabolism of certain amino acids, there is often only one very specific biomarker compound or several closely related compounds (from the same chemical class) detectable in the blood or expelled air that is indicative of a particular metabolic disease or disorder. This specific biomarker is usually the toxic metabolite that accumulates as a result of the enzyme deficiency. Some examples of single-compound biomarker indicators of specific metabolic-related diseases and microbial infections are presented in Table 1.

**Table 1:** Biomarker compounds in the human breath that appear to be uniquely associated with the presence of specific diseases, genetic disorders or adverse physiological conditions in the body.

Biomarker Compounds ^1^	Chemical Class	Associated Diseases/Disorders/Conditions ^2^	References
2,3-butanedione	diketone	URTI	[37]
2-butenal	aldehyde	URTI	[37]
2-butene	alkene	URTI	[37]
2-imidazoleacetic acid	imidazole	Histidinemia	[38]
2-imidazolelactic acid	imidazole	Histidinemia	[38]
2-imidazolepyruvic acid	imidazole	Histidinemia	[38]
2-oxoisocaproic acid	carboxylic acid	BCKD	[38]
2-pentylfuran	furan derivative	Aspergillosis (invasive)	[39]
cadaverine	diamine	Cystinuria	[38,40]
ethylbenzene	benzene derivative	Hyperglycemia	[41,42]
formaldehyde	aldehyde	PLC	[43]
methyl methacrylate	methyl ester	URTI	[37]
methyl nicotinate	methyl ester	Tuberculosis	[44]
phenylacetic acid	benzene derivative	Phenylketonuria	[38]
phenyllactic acid	benzene derivative	Phenylketonuria	[38]
phenylpyruvic acid	benzene derivative	Phenylketonuria	[38]
p-hydroxyphenylpyruvic acid	benzene derivative	Tyrosinemia	[38]
piperidine	heterocyclic amine	Cystinuria	[38,40]
putrescine	diamine	Cystinuria	[38,40]
pyrrolidine	heterocyclic amine	Cystinuria	[38,40]
trans-3-methyl-2 hexenoic acid	fatty acid	SFS	[45]
vinyl butyrate	vinyl ester	URTI	[37]
xylene	dimethyl benzene	Hyperglycemia	[41,42]

^1^ Bio-indicator compounds listed are volatile organic compounds that have been associated with specific diseases, metabolic disorders or adverse physiological conditions, but further independent research may be required to determine the strength of the correlation based on the incidence of these bio-indicator VOCs in the human breath relative to the presence of the indicated ailments. ^2^ Disease abbreviations: BCKD = branched-chain ketoaciduria disorder (maple syrup urine disease); PLC = primary lung cancer; SFS = sweaty feet syndrome; URTI = upper respiratory tract infection.

Other types of single metabolite-specific diseases also exist that are not caused by enzyme deficiencies, but hormone deficiencies, such as hyperglycemia (related to diabetes) and sweaty feet syndrome (SFS), that are correlated with specific biomarkers. Microbial infections, including upper respiratory tract infections (URTI), invasive aspergillosis and tuberculosis (caused by *Mycobacterium tuberculosis* infections), also have been related to the presence of single specific biomarkers.

### 3.2. Biomarkers of Infectious Diseases

A multitude of breath volatile metabolites have been identified as potential biomarkers of many infectious diseases and for cancer detection [8]. Some of these biomarkers associated with more than one infectious disease are listed in Table 2. The association of a specific biomarker to several types of diseases suggests that there may be some commonality to the effects of pathogenesis (associated with different diseases) that may result in similar alterations in biochemical pathways within the body to produce the same abnormal volatile metabolites in common between different and unrelated diseases. In some cases, the pathogenic determinants that are most responsible for causing the diseased condition may be similar (such as enzymes, toxins, *etc*.), resulting in very similar modes of action that alter human metabolic processes to produce identical biomarker metabolites.

**Table 2:** Volatile biomarker compounds in the human breath that are associated with the presence of multiple specific diseases, genetic disorders or adverse physiological conditions in the body.

Biomarker Compounds ^1^	Chemical Structure	Associated Diseases/Disorders/Conditions ^2^	References
Acetaldehyde	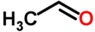	AFDL	[46]
		URTI	[37]
Acetoin(3-hydroxy-2-butanone)	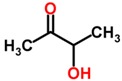	Lung cancer	[47]
		NSCLC	[47]
Acetone	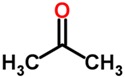	ARDS	[48,49]
		Lung cancer	[50]
		CIP	[48]
		CPD	[51]
		Cystic fibrosis	[52]
		Diabetes mellitus	[41,53]
		Hepatic cirrhosis	[54]
		Ketosis, starvation	[55]
		PLC	[43]
Alkanes, short-chain HC(e.g., ethane)	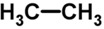	AHI	[56]
		Asthma	[57,58,59]
		COPD	[60]
		Cystic fibrosis	[52,61]
		IBD	[62,63,64,65]
		IHD, angina	[66,67]
		ILD	[68,69]
		Lung cancer	[66,70,71,72]
		Oxidative stress	[73]
		Schizophrenia	[74,75,76]
1-Butanol	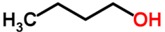	Lung cancer	[47]
		NSCLC	[47]
Carbon disulfide	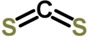	Cystic fibrosis	[52,77]
		Schizophrenia	[74,75,76]
Dimethyl sulfide	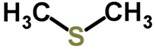	Lung cancer	[78]
		Chronic hepatitis	[79]
		Cystic fibrosis	[52,77]
		Endocarditis (infective)	[80,81,82,83]
		Hepatic cirrhosis	[79,84,85,86]
		Hepatic coma	[79,86, 87]
Ethanol	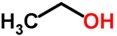	CPD	[51]
		Cystic fibrosis	[52]
		Diabetes mellitus	[41,53]
Hydrogen sulfide	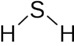	Endocarditis (infective)	[80,81,82,83]
		Hepatic cirrhosis	[84]
Isoprene	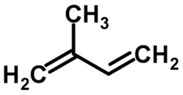	AFDL	[46]
		ARDS	[48,49]
		Asthma	[57,58,59]
		CIP	[48]
		Cystic fibrosis	[52,88]
		Lung cancer	[50]
		PLC	[43]
8-Isoprostane(8β-prostane)	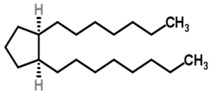	Asthma	[89]
		COPD	[90]
		Oxidative stress	[89]
Leukotriene B4	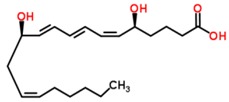	Asthma	[91,92]
		COPD	[90]
		Cystic fibrosis	[93]
Methanol	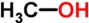	Cystic fibrosis	[52]
		Lung cancer	[50]
Methyl nitrate	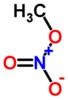	Diabetes mellitus	[41,53]
		Hyperglycemia	[41,42]
Methylated alkanes(e.g., 2-methylpropane)	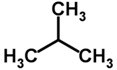	Breast cancer	[94]
		IHD, angina	[66,67]
		Lung cancer	[66,70]
		Oxidative stress	[73]
Methyl-mercaptan	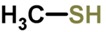	Chronic hepatitis	[79]
		Endocarditis (infective)	[80,81,82,83]
		Hepatic cirrhosis	[79,86]
		Hepatic coma	[79,86,87]
Nitric oxide	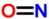	Asthma	[95]
		COPD	[96]
Nitrotyrosine	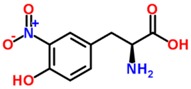	COPD	[97]
		Cystic fibrosis	[97]
Pentane	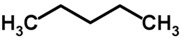	AHI	[56]
		ARDS	[48,49]
		Asthma	[57,58,59]
		CIP	[48]
		Cystic fibrosis	[52]
		IBD	[62,63,64,65]
		Lung cancer	[98]
		Rheumatoid arthritis	[99]
		Schizophrenia	[74,75,76]
Propane	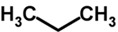	Cystic fibrosis	[52]
		IBD	[62,63,64,65]
o-Toluidine	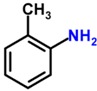	Lung cancer	[100]
		PLC	[43]

^1^ Biomarker compounds listed are VOCs that have been associated with specific diseases, metabolic disorders or adverse physiological conditions, although further independent research may be required to determine the strength of the correlation based on the incidence of these biomarker VOCs in the human breath relative to the presence of the indicated ailments. ^2^ Disease abbreviations: AFDL = alcoholic fatty liver disease; AHI = alcohol-induced hepatic injury; ARDS = acute respiratory stress syndrome; CIP = critically ill patients; CPD = cardiopulmonary disease; COPD = chronic obstructive pulmonary disease; IBD = inflammatory bowel disease; IHD = ischemic heart disease; ILD = interstitial lung disease (includes cryptogenic organizing pneumonia, idiopathic pulmonary fibrosis, sarcoidosis, *etc*.); PLC = primary lung cancer; NSCLC = non-small cell lung cancer; URTI = upper respiratory tract infection.

Certain volatile biomarkers, such as acetone, certain alkanes, dimethyl sulfide, isoprene, methyl mercaptan and, pentane tend to be produced in response to a number of different diseases, suggesting a commonality of effects on the same human metabolic processes by virtue of the fact that only certain metabolic pathways are responsible for producing each of these biomarkers. As a result, the presence of these specific biomarkers in the human breath may be good general indicators of a diseased condition in the body and prompt further diagnostic investigations to find out the specific disease involved among those most associated with particular biomarkers.

## 4. Applications of Biomarker Detection in Disease Diagnosis

The applied science area involving the use of metabolite biomarkers (metabolomics) in disease detection and diagnosis is still at an early stage of development; however, this approach is gaining increasing interest and will likely continue to progress toward developing standardized diagnostic methods for point-of-care clinical applications using e-nose-type devices that sense complex mixtures of abnormal volatile biomarkers in the breath of patients. Progress toward this goal will be facilitated by the identification and confirmation of new breath biomarker mixtures, defined as specific electronic breath signature patterns, as strong indicators of specific diseases in the body.

### 4.1. Conventional Methods of Biomarker Detection

Traditional analytical methods for the detection and identification of VOCs in metabolomic studies, including such instruments as gas chromatography-mass spectroscopy (GC-MS), nuclear magnetic resonance (NMR) and infrared spectroscopy (IR), are now supported by other new analytical methods. Human breath analysis now employs newer devices to identify breath biomarkers, including proton transfer reaction MS (PTRMS) [101], selected ion flow tube mass spectrometry (SIFT-MS) [102] and high-sensitivity laser spectroscopic techniques, including tunable diode laser absorption spectroscopy (TDLAS), cavity ringdown spectroscopy (CRDS), integrated cavity output spectroscopy (ICOS), cavity-enhanced absorption spectroscopy (CEAS), cavity leak-out spectroscopy (CALOS), photoacoustic spectroscopy (PAS), quartz-enhanced photoacoustic spectroscopy (QEPAS) and optical frequency comb cavity-enhanced absorption spectroscopy (OFC-CEAS) [103]. Unfortunately, these time-consuming and very technical analytical methods are useful only for identifying individual compounds in breath metabolite mixtures, but are not so useful for identifying complex breath mixtures (breath sample signatures) as a whole that may contain many biomarkers of disease. These are the reasons why simpler, easier to use electronic devices, such as e-noses, will likely be used in the future to simultaneously detect complex mixtures of breath biomarkers more useful for diagnostic disease detection.

### 4.2. Electronic-Nose Technologies for Biomarker Detection

The utilization of e-nose instruments in metabolomics primarily involves the detection of complex volatile mixtures of biomarkers in the breath, which are analyzed as digital breath patterns that can be recognized via application-specific databases and correlated with the presence of specific diseases in associated organs or compartments of the body. Some examples where e-nose devices have been utilized in various studies conducted in research hospitals and universities worldwide to detect specific lung diseases from breath analyses are presented in Table 3. Most of these studies have involved large numbers of patients, several different e-nose instrument types and variable numbers of sensors in the sensor arrays for the detection of some important lung and heart diseases, including acute respiratory stress syndrome (ARDS), asthma, cancer, chronic obstructive pulmonary disease (COPD), endocarditis, malignant pleural mesothelioma (MPM), pulmonary tuberculosis (TB), upper respiratory tract infections (URTI) and ventilator-associated pneumonia (VAP). In most cases, e-nose devices have been quite effective at detecting specific diseases from breath analyses and at distinguishing between different diseases detected based on differences in breath signature patterns resulting from variations in volatile biomarker compounds found in patients’ breath samples.

**Table 3:** Studies utilizing electronic-nose instruments to detect aroma profiles containing volatile biomarker compounds as indicators of disease in the human breath, exhaled breath condensate, bronchi, or alveolar air.

Disease Detection/Discrimination ^1^	Organ	*N*^2^	Study Locations ^3^	E-Nose Type/No. Sensors ^4^	References
ARDS-MPM	Lung	78	St. Vincent and Price of Wales Hospital, Sydney, Australia	CP 32	[104]
Asthma	Lung	40	Leiden University MC, Leiden, the Netherlands	CP 32	[105]
		51	Instituto Dermopatico deli’ Immacolata, Rome, Italy	QMB 8	[106]
Asthma-COPD	Lung	90	Academic MC Amsterdam, Haga Teaching Hospital, The Hague; Albert Schweitzer Hospital, Dordrech, the Netherlands	CP 32	[107]
		100	Academic MC Amsterdam, Haga Teaching Hospital, The Hague; Albert Schweitzer Hospital, Dordrech, the Netherlands	CP 32	[108]
		44	University of New South Wales, Sydney, Australia	CP 32	[109]
Cancer	Lung	62	C. Forlanini Hospital, Rome, Italy	QMB 8	[71]
		135	Cleveland Clinic, Ohio, USA	CP 32	[110]
		143	Cleveland Clinic, Ohio, USA	CM 36	[111]
		92	C. Forlanini Hospital, Rome, Italy	QMB 8	[112]
		229	Cleveland Clinic, Ohio, USA	CM 36	[113]
COPD	Lung	43	Phillipps University, Marburg, Germany	CP 32	[114]
COPD-Cancer	Lung	30	Leiden University MC, Leiden, the Netherlands	CP 32	[115]
Endocarditis (infective)	Heart	78	Osaka University, Osaka, Japan.	MOS 6	[116]
IPA	Lung	53	University of Amsterdam, Amsterdam, The Netherlands	CP 32	[117]
MPM	Lung	39	University of Bari Aldo Moro, Bari, Italy	CP 32	[118]
Pneumonia	Lung	400	Dept. of Anesthesia, University of Pennsylvania, Philadelphia, PA, USA	BS	[119]
TB	Lung	46	Cranfield University, Silsoe, Bedfordshire, UK	CP 14	[120]
		134	Cranfield University, Silsoe, Bedfordshire, UK	CP 14	[121]
		279	University of Santo Tomas, Manila, Philippines; De La Salle University Hospital, Cavite, Philippines; Homerton University Hospital, London, UK; Hinduja Hospital, Mumbai, India	SAW 1	[122]
URTI	Respiratory tract	NS	University of Pennsylvania Medical Center, Philadelphia, Pennsylvania, USA	CP 32	[123]
VAP	Lung	25	University of Pennsylvania, Philadelphia, Pennsylvania, USA	CP 32	[124]
		38	University of Pennsylvania, Philadelphia, Pennsylvania, USA	CP 32	[119]
		44	University of Pennsylvania, Philadelphia, Pennsylvania, USA	CP 32	[125]

^1^ Abbreviations: ARDS = Acute Respiratory Stress Syndrome; COPD = Chronic Obstructive Pulmonary Disease; IPA = Invasive Pulmonary Aspergillosis; MPM = Malignant Pleural Mesothelioma; TB = pulmonary Tuberculosis; URTI Upper Respiratory Tract Infections); VAP = Ventilator Associated Pneumonia; ^2^ Sample size collected from individual patients; NS = not specified; ^3^ Hospital or university location where the study was conducted; ^4^ Electronic-nose instrument type and number of sensors utilized in the e-nose sensor array for detection of lung diseases through analysis of human-breath signature patterns. BS = biosensor (experimental technology); CM = calorimetric; CP = conducting polymer; MOS = metal oxide semiconductors; QMB = quartz microbalance; SAW = surface acoustic wave.

The electronic nose devices used in the experimental and clinical trials of the studies listed here were predominately conducted with polymer-type e-noses with 32 sensors in the sensor array. Conducting polymers are low power-consuming instruments with very good sensitivity and reproducibility (precision) at near room temperatures, but they have some disadvantages, including high sensor sensitivity to moisture, inactivation by certain strongly-polar analytes and relatively low sensor life (compared to MOS sensors) that tend to have a much longer sensor life, but operate at higher temperatures and with greater power demand. CP-sensors and some types of MOS sensors also sometimes exhibit problems of sensor compatibility and uniformity. QMB and SAW sensors have very good sensitivity, but are not as easily effective for the development of breathprints that can be stored in reference databases for comparisons and analysis of new sample unknowns, primarily due to the lack of available statistical software utilities with pattern recognition algorithms.

## 5. Conclusions and Future Prospects

The human breath is an obvious medium for point-in-time or continuous sampling and analysis of VOCs generated within the body, because these volatile compounds travel through the body via the circulatory system in the blood, cross the alveolar interface and are released in exhaled breath [126]. Analysis of VOCs in the breath can provide an indicator of current metabolic status for establishing a patient’s level of health and the existence of any diseased states. E-nose technologies have the capabilities of non-invasively and painlessly detecting diseases in their early stages, allowing for early treatments and usually more rapid recovery of patients from diseased states. Thus, the VOCs present in the exhaled breath are a potentially rich and powerful source of biomarkers for the effective e-nose detection and diagnosis of not only respiratory diseases, but also many other diseases in the body.

Breath fingerprint analysis, coupling the use of traditional chemical analysis methods to identify metabolites with new EAD technologies, offers promising progress with great potential for promoting non-invasive methods of diagnosing the disease states of clinical patients. New e-nose methods and algorithms to analyze complex mixtures of breath VOCs offer high sensitivity, accuracy, precision, low response time and low detection limits, which are desirable characteristics for the detection of VOCs in human breath. The need for the standardization of breath sample collection and analysis methods are among the key issues thwarting the full-scale introduction of breath tests into clinical practice [127].

The future outlook for continued progress in the improvement of biomarker breath analysis to facilitate the accuracy and effectiveness of disease diagnostics is encouraging given all of the potential advantages afforded by the continued development of new e-nose technologies to detect the many and varied metabolic breath signatures associated with specific diseases found in different compartments and organs of the human body. The popularity and global enthusiastic interests in the noninvasive “biomarker” approach to disease diagnostics, based on gas analysis of the human breath, will no doubt lead to great new strides and accomplishments as new biomarkers and biomarker mixtures are discovered and more firmly correlated with specific diseases localized in various organs of the body. Very recent research and reviews have confirmed that e-noses are effective at recognizing VOC profiles, such as in disease-specific breath-prints, to accurately diagnose different pulmonary diseases, including ARDS [128], cystic fibrosis (CF), COPD [129], VAP [130], asthma, lung cancer [131] and various respiratory tract infections [132].

The desired combination of the e-nose capabilities of specificity, selectivity, robustness in operation, reproducible sensor uniformity and long-life stability is not offered by current e-nose instruments for most disease-diagnostic applications [133]. These technologies currently are unable to identify individual compounds, although they may be used to compare samples to see whether they have similar VOC profiles. VOC profiles or breathprints may be sufficient for diagnostic purposes in some respiratory diseases without having to identify individual compounds present in exhaled breath.

There is a need to build world-wide diagnostic biomarker databases for disease diagnosis, and associated clinical applications will facilitate the use of e-nose technologies for diagnostic applications in universal healthcare facilities, as well as for remote care applications using portable e-nose devices coupled with satellite uplinks and wireless Internet communication systems in order to access healthcare professionals in hospitals.

The need for dedicated application-specific e-noses combined with specialized databases for the diagnosis of specific disease categories should significantly improve and standardize the methods used by physicians to diagnose specific types and categories of diseases based on VOCs in patients’ breath.

Future improvements of e-nose instruments, allowing the identification of biomarkers in breathprints, via interfacing e-noses in tandem with mass spectrometers or other analytical devices, may facilitate disease diagnoses in some cases, although better correlations of diagnostic disease biomarker mixtures in unique breathprints to specific diseases will go a long way toward making the use of e-nose technologies a well-established reality in point-of-care clinical diagnosis.

Miniaturization of e-nose technologies using fewer sensors, smart selection of sensor types used in the sensor array and improved pattern recognition algorithms designed specifically to recognize unique breathprints, based on the presence of disease-specific mixtures of biomarkers, so that analyses can become more accurate, dependable and unequivocal, results in delivering effective early diagnoses of organ-specific diseases from breath analysis of patients during routine annual noninvasive checkups involving simple breath tests, rather than invasive biopsies or painful procedures, such as mammograms and colonoscopies.
